# Dosage Considerations for Transcranial Direct Current Stimulation in Children: A Computational Modeling Study

**DOI:** 10.1371/journal.pone.0076112

**Published:** 2013-09-27

**Authors:** Sudha Kilaru Kessler, Preet Minhas, Adam J. Woods, Alyssa Rosen, Casey Gorman, Marom Bikson

**Affiliations:** 1 Children’s Hospital of Philadelphia and the Perelman School of Medicine at the University of Pennsylvania, Philadelphia, Pennsylvania, United States of America; 2 The City College of the City University of New York, New York, New York, United States of America; 3 Center for Cognitive Neuroscience, University of Pennsylvania, Philadelphia, Pennsylvania, United States of America; Cardiff University, United Kingdom

## Abstract

Transcranial direct current stimulation (tDCS) is being widely investigated in adults as a therapeutic modality for brain disorders involving abnormal cortical excitability or disordered network activity. Interest is also growing in studying tDCS in children. Limited empirical studies in children suggest that tDCS is well tolerated and may have a similar safety profile as in adults. However, in electrotherapy as in pharmacotherapy, dose selection in children requires special attention, and simple extrapolation from adult studies may be inadequate. Critical aspects of dose adjustment include 1) differences in neurophysiology and disease, and 2) variation in brain electric fields for a specified dose due to gross anatomical differences between children and adults. In this study, we used high-resolution MRI derived finite element modeling simulations of two healthy children, ages 8 years and 12 years, and three healthy adults with varying head size to compare differences in electric field intensity and distribution. Multiple conventional and high-definition tDCS montages were tested. Our results suggest that on average, children will be exposed to higher peak electrical fields for a given applied current intensity than adults, but there is likely to be overlap between adults with smaller head size and children. In addition, exposure is montage specific. Variations in peak electrical fields were seen between the two pediatric models, despite comparable head size, suggesting that the relationship between neuroanatomic factors and bioavailable current dose is not trivial. In conclusion, caution is advised in using higher tDCS doses in children until 1) further modeling studies in a larger group shed light on the range of exposure possible by applied dose and age and 2) further studies correlate bioavailable dose estimates from modeling studies with empirically tested physiologic effects, such as modulation of motor evoked potentials after stimulation.

## Introduction

Transcranial direct current stimulation (tDCS) is an emerging technology for non-invasive modulation of neural activity using weak electrical currents applied to the scalp. By imposing a voltage gradient on cortical neurons along the path of current, direct current stimulation induces regional changes in cortical excitability that persist beyond the stimulation period [1]. The effects of tDCS depend on the intensity and spatial extent of induced electrical fields, which in turn depend on both the stimulation dose (applied current intensity, arrangement of electrodes) and neuroanatomic factors [2]. TDCS is being actively investigated as a therapeutic tool for a number of neurologic and psychiatric conditions, including epilepsy and rehabilitation from injury, conditions which affect children as well as adults [3]. While the investigational use of tDCS in adults is widespread, information on the use of tDCS in children is limited. It is unclear whether dose parameters considered safe and efficacious for use in adults should be adjusted to achieve comparable results in children.

Density and spatial distribution of current in the brain have critical implications for tDCS safety and efficacy. Because the tissue structure of the skull and its contents is different in children compared with adults, understanding how these differences might affect current flow from the electrodes to the brain is necessary for designing rational tDCS protocols in children. In addition to age dependent differences in gray and white matter content and CSF volume [4], there is also a developmental trajectory for brain-scalp distance [5] – factors which may affect peak current densities at the skin, the brain surface, and sub-cortical regions. MRI-derived finite element models used to map electrical fields produced by tDCS have shown that tissue architecture critically influences current flow [6–10]. The objective of this study was to use pediatric MRI-derived head models to gain insight into safety issues and to optimize the design of pediatric tDCS investigations.

## Methods

Individualized MRI-derived models of three healthy adults (2 females (33 and 25 years, respectively) – later referenced as S1 and S2, and 1 male (36 years – referenced as S3) and two typically developing, neurologically normal male children (ages 8 and 12 – later referenced as P1 and P2, respectively) were developed using methods previously described [6,11]. Written exemption from review was issued by the institutional review board (IRB) of the City College of New York, where this study was performed, on the determination that computational modeling studies using de-identified MRI images is not human subjects research, consistent with 45 CFR 46.101(b)(4) of the U.S. Health and Human Services regulations. MRI images of the two children were obtained from a repository of images collected for other studies approved by the Children’s Hospital of Philadelphia Institutional Review Board. Written consent for storage in a hospital database and future use of de-identified images was explicitly given by parents/guardians of subjects on their behalf at the time the scans were obtained. MRI data for P1 and P2 were acquired on a 3T Siemens VerioTM scanner, and each subject had a 3D MP-RAGE anatomic scan in the axial orientation, with a field of view of 256X256X192 and matrix 256x256x192 to yield 1 mm^3^ isotropic voxel resolution. MRI data for S1 were obtained using a 3T General Electric, Signa Excite HD scanner (Fairfield, CT). The T1-weighted images were acquired using a GRE sequence with TE = 2.2 ms, TR=7.3 ms, 256 x 256 matrix scan with 212 axial slices and an isotropic resolution of 1 mm^3^. S2 was scanned at the center for Biomedical Imaging Boston University School of Medicine, using a 3-T Philips Achieva scanner (Phillips Medical Systems, Best, Netherlands). Acquisition parameters were: TE = 3.2 ms; TR= 6.92 ms; flip angle = 82°; FOV=256x256 mm; resolution= 256x 256; slice thickness = 1.2 mm; no gap; and voxel size of 1 x 1 x 1.2mm. S3 was scanned on a 3T Siemens Trio scanner (Erlangen, Germany). The T1-weighted images were collected using gradient echo (GRE) sequence with TE= 2.3 ms, TR= 1900 ms, 280 x 320 matrix scan with 208 sagittal slices and had a isotropic resolution of 1 mm^3^.

**Figure 1:**
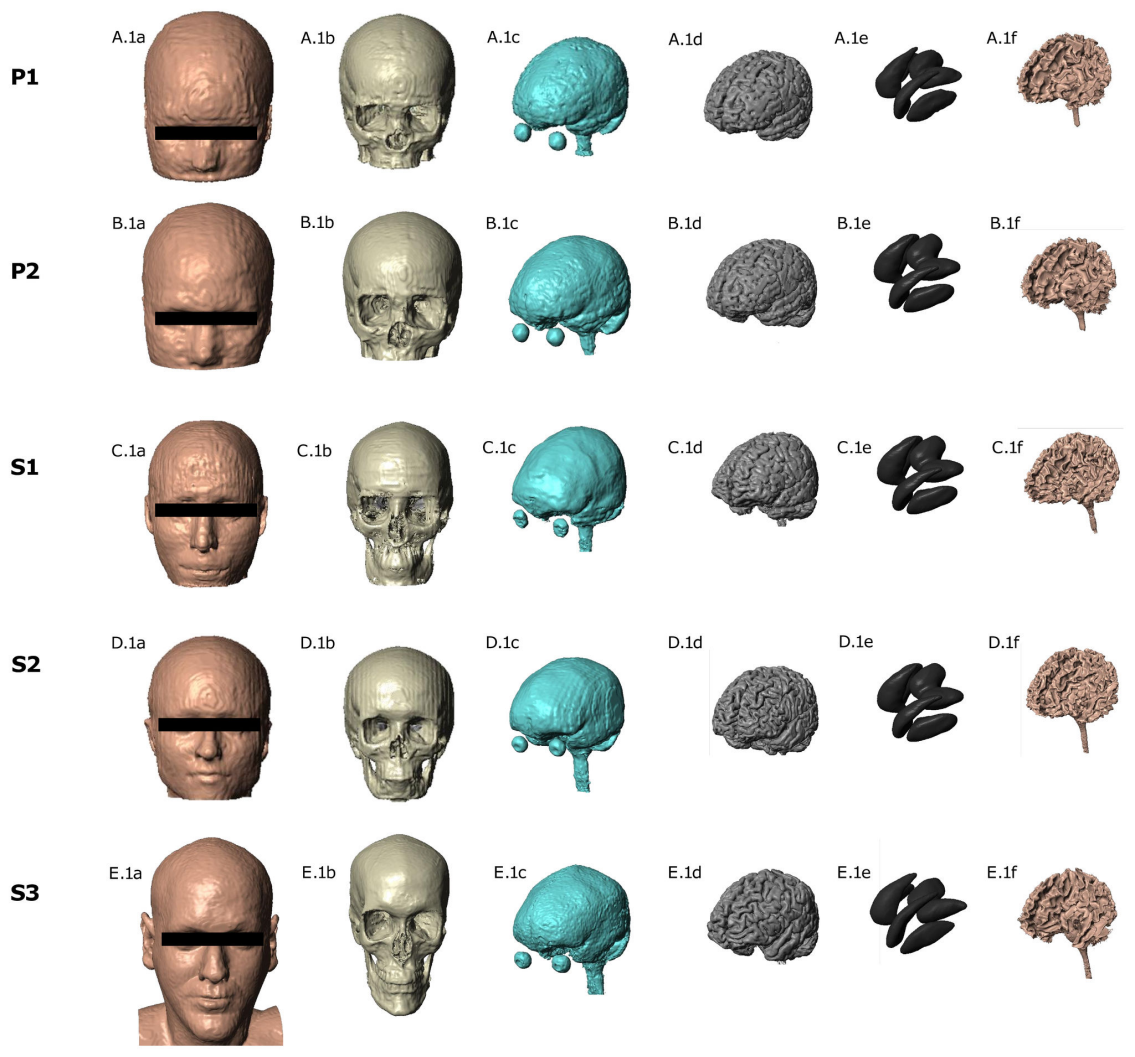
Segmented structures. Segmented tissue masks (skin, skull, CSF, gray matter, and white matter respectively) for the two children (P1, P2) and three adults (S1,S2,S3).

Scans were segmented into eight masks (soft tissue- including skin, fat, and muscle; bone; air; eyes; cerebrospinal fluid- including macroscopic brain blood vessels; cortical gray matter; white matter; and deep gray matter) using a combination of automated methods (FSL, FMRIB Analysis Group, Oxford, UK) and manual segmentation tools (Simpleware Ltd, Exeter, UK) (Figure 1). Stimulation pads, disk electrodes, and gel were rendered as CAD files (.stl) and imported into ScanCAD (Simpleware Ltd, Exeter, UK) for manual positioning over the scalp of the 3D model. The finite element adaptive meshes generated from the segmentation and CAD masks, consisting of >5,000,000 tetrahedral elements (>9,000,000 degrees of freedom) were imported into COMSOL Multiphysics 3.5 (Comsol Inc,MA).

The model was solved using a linear system solver of conjugate gradients with a relative tolerance of 1x10^6^. The electrical properties of tissues were defined by the average isotropic conductivity (S/m): cortical and deep gray matter 0.276 S/m; white matter: 0.126 S/m; CSF 1.65 S/m; bone 0.01 S/m, eyes 0.4 S/m, scalp with fat and muscle tissue 0.465 S/m).

We modeled six exemplary electrode configurations. Four configurations utilized conventional 25 cm^2^ sponge-based anode and cathode electrodes. A conventional electrode consists of a rubber electrode covered in a rectangular sponge, which is soaked in saline for application of tDCS. Sponges were therefore assigned the conductivity of saline (1.4 S/m). Two configurations consisted of a high definition tDCS (HD-tDCS) montage using 11 mm diameter disc electrodes submerged in electrode gel(0.3 S/m conductivity). For the HD-tDCS montages, four cathode disc electrodes were arranged around an anode center electrode. All electrodes had a thickness of 1mm and were modeled as conductors with the conductivity of copper 5.8 x 10^7^ S/m. The thickness of the gel was 2 mm. The thickness of the sponge varied from 1 to 2.5 mm, changing with scalp curvature to maintain continuous contact. The five configurations used were:

1 Lateralized Motor: The anode was placed over the left primary motor cortex with its center over the C3 position in the international 10-20 system. The cathode was placed over the corresponding position on the right motor cortex (centered over the C4 position).
2 Lateralized Temporal: The anode was centered over the posterior aspect of the superior temporal gyrus of the left hemisphere, and the cathode was centered over the analogous location in the right hemisphere.
3 Lateralized Dorsolateral Prefrontal: The anode was centered over the left dorsolateral prefrontal cortex with its center over the F3 position in the international 10-20 system. The cathode was placed over the corresponding position on the right motor cortex (centered over the F4) position.
4 M1-SO: The anode was placed over the left primary motor cortex with its center over C3, and the cathode was placed over the contralateral supraorbital area.
5 x1 HD-tDCS ring, wide: The anode was placed over the C3 position, in left primary motor cortex, with cathode electrodes arranged in a circular fashion around the anode, with a disc-center to disc-center radius of 5 cm. This distance corresponds to the distance between electrode sites using the international 10-20 EEG system in a typical adult.
6 x1 HD-tDCS ring, narrow: This configuration was the same as the wide HD-tDCS configuration described above, except with a smaller ring (disc-center to disc-center radius of 2.5 cm). The smaller ring configuration was modeled only for the pediatric heads. Because the circumference of an adult head is larger than that of a child, the smaller distance corresponds more closely to the distance between electrode sites using the international 10-20 system in a typical 8-12 year old child.

The Laplace equation ∇(σ∇V)=0 (*V*: potential; σ: conductivity) was solved and the boundary conditions used were (1) inward current flow = J_n_ (normal current density) applied to the exposed surface of the anode electrode (2), ground applied to the exposed surface of the cathode electrode(s) and (3) all other external surfaces treated as insulated. Current densities corresponding to 0.5 mA, 1 mA, and 2 mA were applied.

Plots of electrical field magnitude over the cortical surface, and on coronal cross sectional slices from directly beneath the center of the anode, were generated. As the conductivity of the grey matter in the model is uniform, plots of the control surface also represent current density, where current density values can be scaled using: J= σE. In addition, directionality of current flow was investigated.

Anatomical differences, across heads, were examined. Using tools in the mask generation software (Simpleware Ltd. UK), the following measurements were calculated for each head: Ear-to-ear distance, nasion-to-inion distance, soft tissue thickness and skull thickness. Soft tissue and skull thicknesses were measured over a region approximating the C3 position in the 10-20 system of electrode placement, and was estimated as the total volume of the selected tissue over the surface area of the same region.

## Results

### Anatomical Features

**Figure 2:**
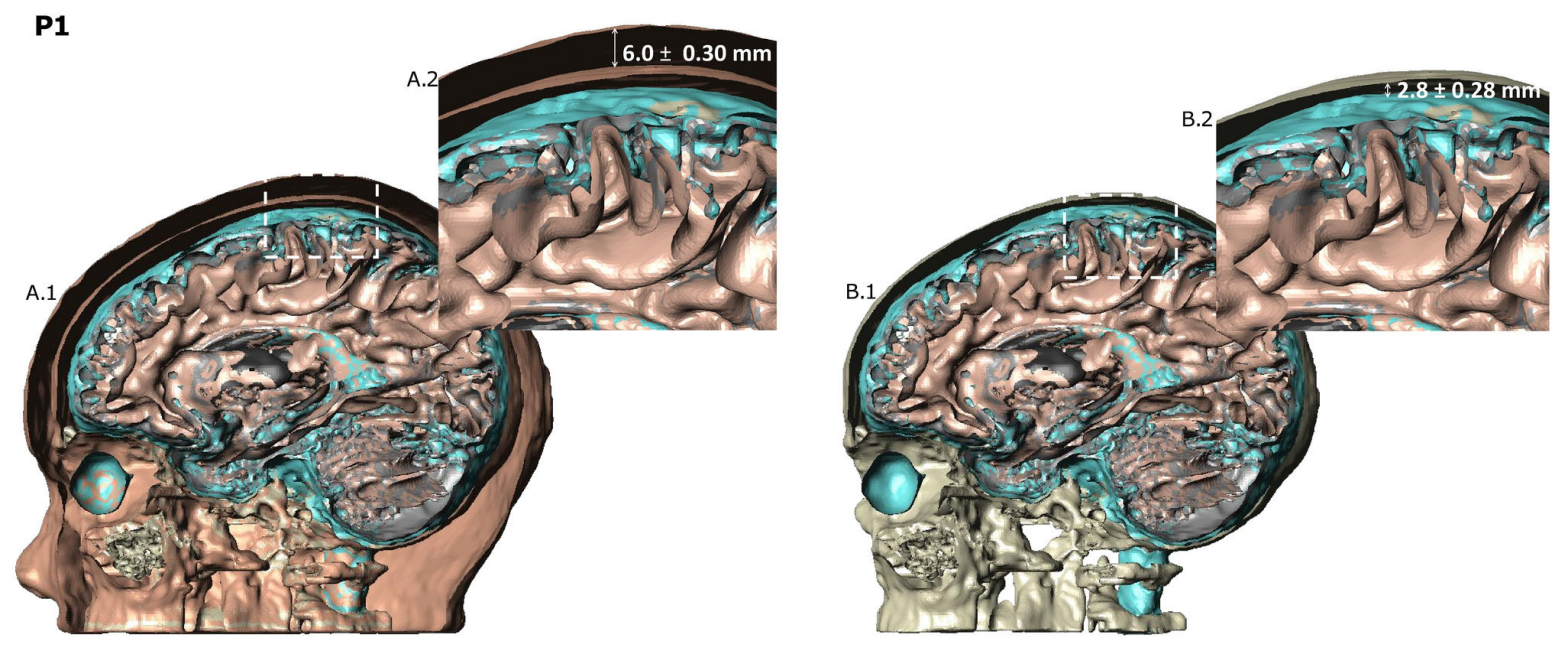
Intervening tissue thickness. Skin and skull thickness are among important factors that determine the flow of current through the brain. The region from which the measurements were taken are shown for the 8 year old case (A1,B1). The skin and skull thickness for the 8 year old was 6.0 ± 0.30 mm and 2.8 ± 0.28 mm, respectively.

Figure 1 illustrates segmented 3-dimensional tissue masks for all five head models. Figure 2 illustrates in one model the location of soft tissue and skull thickness measurements in cross section. Table 1 summarizes soft tissue thickness, skull thickness, ear-to-ear distance, and nasion-to-inion distance. While tissue thickness and head size was greater in S3 than in the pediatric models, measurements in S1 and S2 more closely approximated those of P1 and P2, with notable differences in skull thickness.

**Table 1:** Anatomic parameters for each model estimated from reconstructed 3 dimensional masks.

Model	Skin Thickness (mm)	Skull Thickness (mm)	Ear to Ear length (mm)	Nasion to Inion length (mm)
P1	6.0 ±0.31	2.8±.28	141.4	192.1
P2	5.1±0.41	3.1±0.11	140.3	190.5
S1	5.0±0.30	4.8±0.17	150.9	191.5
S2	4.6±.30	4.6±0.24	150.2	196.5
S3	6.5±0.34	4.3±0.34	161.3	203.7

### Cortical electric field intensity

**Figure 3:**
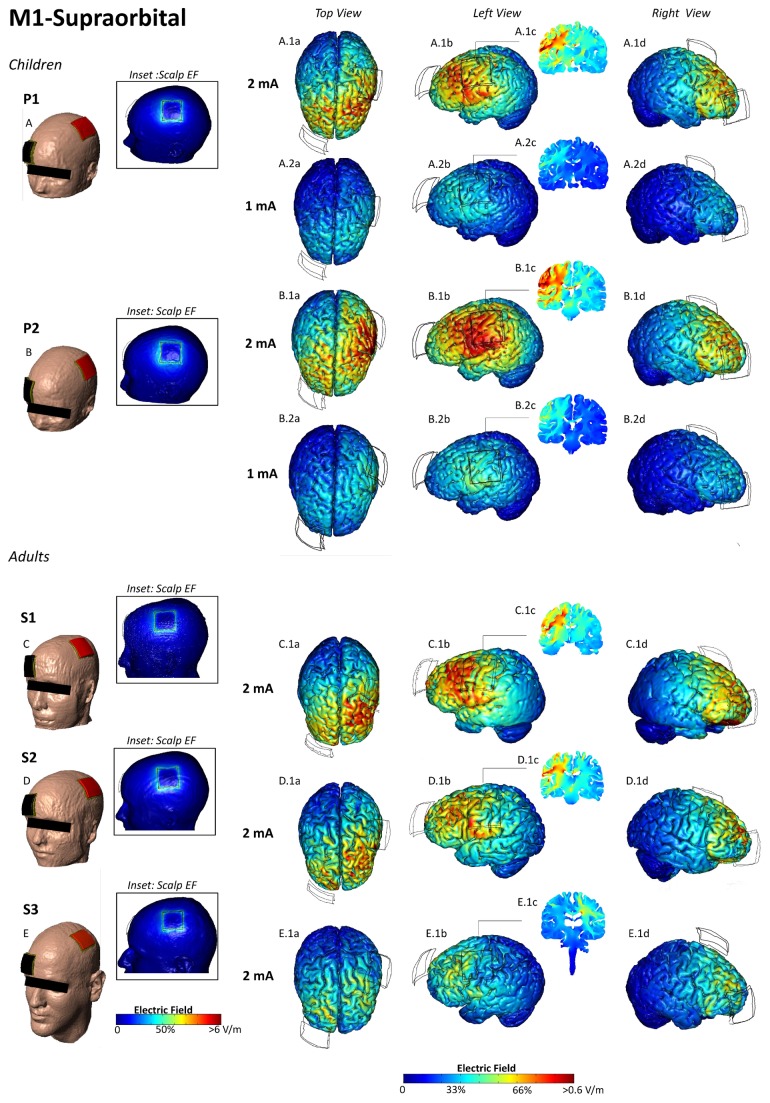
Electric field plots on the cortical surface of the pediatric and adult brains for the M1-SO montage. The center of anode (red) was positioned on the motor strip and the cathode (black) was positioned over the contraletral supraorbital area (A-E). At 2 mA, the peak electric field was 0.66 V/m, 0.88 V/m, 0.72 V/m, 0.58 V/m, 0.56 V/min for P1, P2, S1, S2, and S3 respectively (A.1a,b,d- E.1a,b,d). A.2a,b,d, B.2a,b,d - show EF plots at 1 mA, for the pediatric heads. Cross-sectional coronal electric field plots were taken from the center of the anode (A.1c, A.2c, B.1c, B.2c, C.1c, D.1c, E.1c).

**Figure 4:**
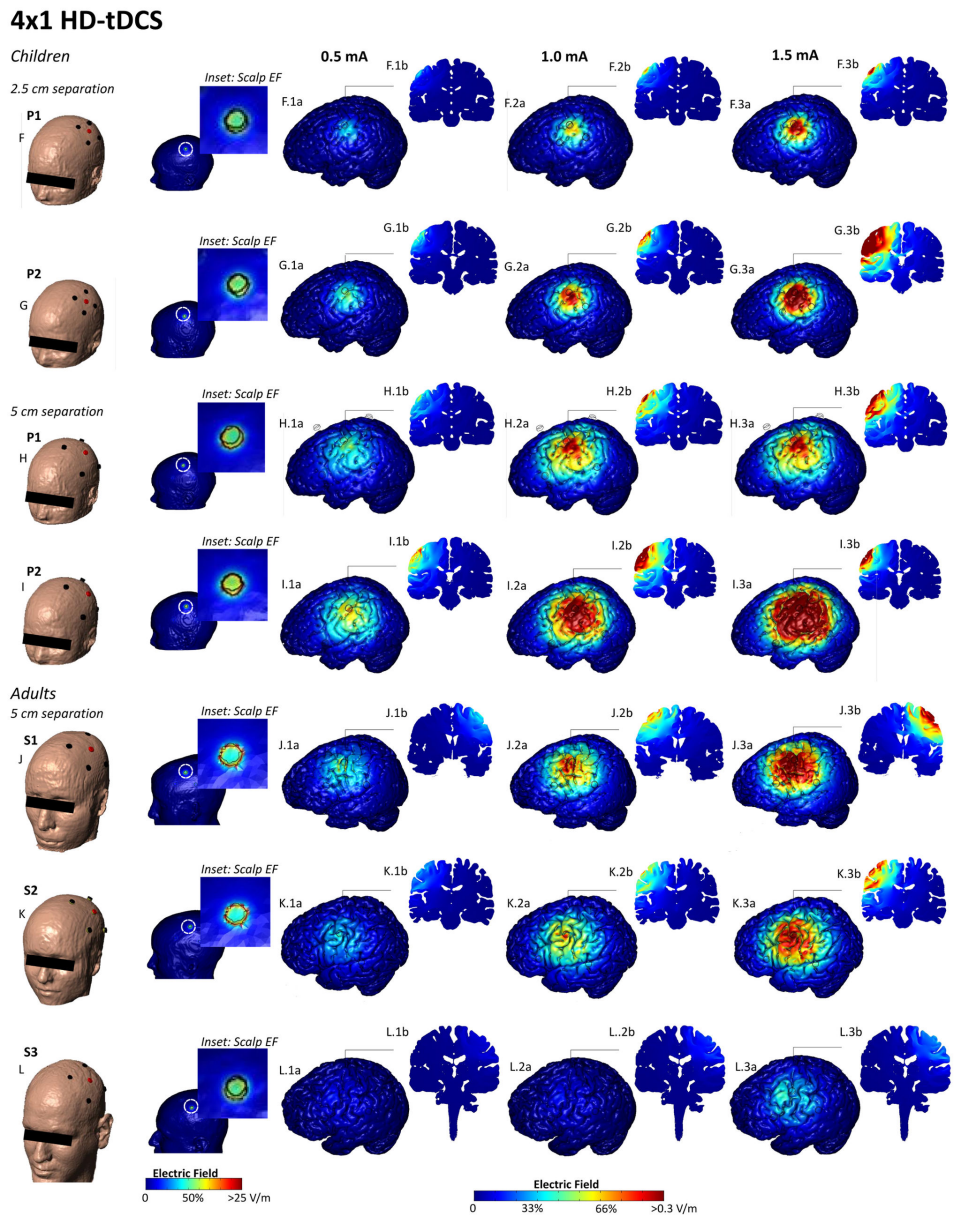
Electric field plots on the cortical surface of the pediatric and adult brains for HD-tDCS montage. For 4x1 high definition tDCS the center of the anode (red) was positioned on the motor strip and the four returns (black) were placed around the center in a circular fashion with a 5cm distance from the center of the anode to the center of the return (F-L). The peak electric field, at 2 mA, for 4x1 HD-tDCS was 0.68 V/m, 0.90 V/m, 0.88 V/m, 0.48 V/m, and 0.22 V/m in P1, P2, S1, S2, and S3, respectively, at a 5 cm separation (center of anode to center of cathode distance). An additional smaller ring (2.5 cm separation) was modeled for the adolescents (see methods) (G, I). At a 2.5 cm separation, the peak electric field was 0.42 V/m and 0.68 V/m, for P1 and P2 respectively. False color maps of 0.5 mA, 1 mA, and 1.5 mA of current are shown, respectively, in the adolescents and adults (F.1-3a, G.1-3a, H.1-3a, I.1-3a, J.1-3a, K.1-3a, L.1-3a). Cross-sectional coronal electric field plots were taken from the center of the anode (F.1-3b, G.1-3b, H.1-3b, I.1-3b, J.1-3b, K.1-3b, L.1-3b).

**Figure 5:**
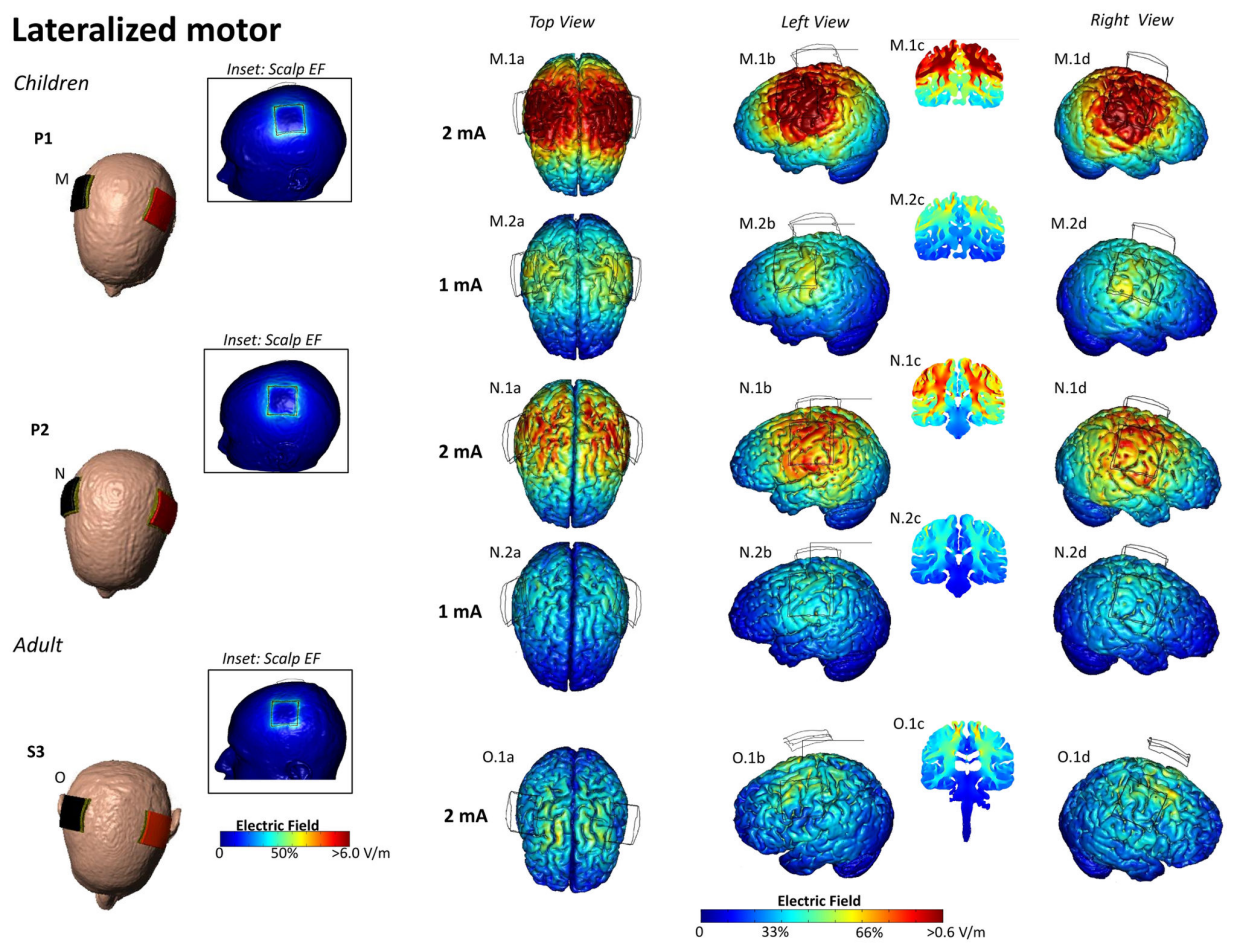
Electric Field plots on the cortical surface of the Pediatric and Adult Brains for Lateralized Motor montage. The center of anode (red) was positioned on the motor strip and the cathode (black) was positioned contralateral to the anode (M-O). At 2 mA, the peak electric field was 0.88 V/m, 0.80 V/m, 0.42 V/m for P1, P2 and S3 respectively (M.1a,b,d- O.1a,b,d). M.2a-d, N. 2a-d – show EF plots at 1 mA, for the pediatric heads. Cross-sectional coronal electric field plots were taken from the center of the anode (M.1c, M.2c, N. 1c, N. 2c, O.1c).

**Figure 6:**
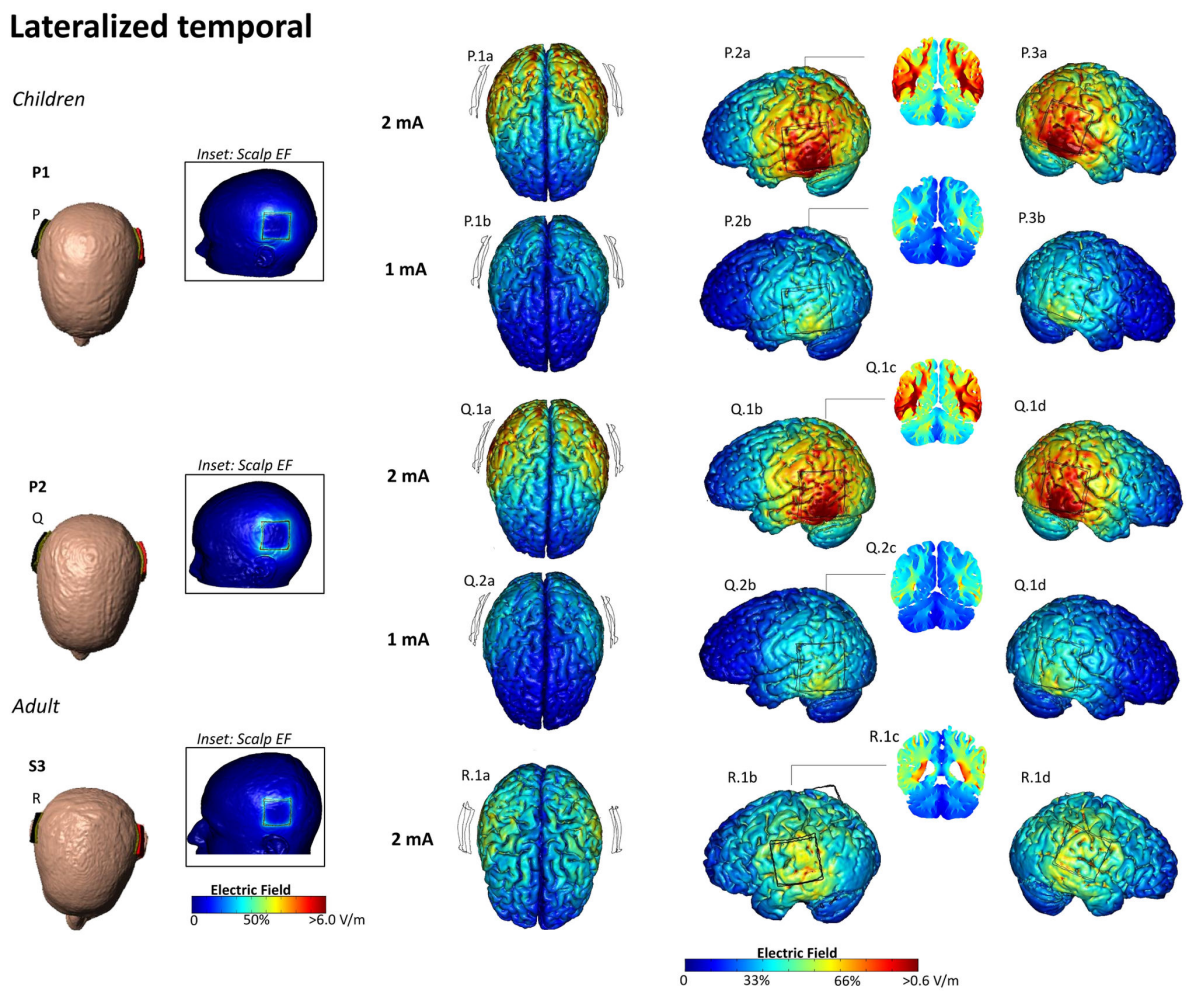
Electric field plots on the cortical surface of the pediatric and adult brains for Lateralized Temporal montage. The center of anode (red) was positioned over the left temporal lobe and the cathode (black) was positioned contralateral to the anode (M-O). At 2 mA, the peak electric field was 1.00 V/m, 0.92 V/m, 0.66 V/m for P1, P2 and S3 respectively (P.1a,b,d- R.1a,b,d). P.2a-d, Q.2a-d - show EF plots at 1 mA, for the pediatric heads. Cross section coronal electric field plots were taken from the center of the anode. Cross-sectional coronal electric field plots were taken from the center of the anode (P.1c, P.2c, Q.1c, Q.2c, R.1c).

**Figure 7:**
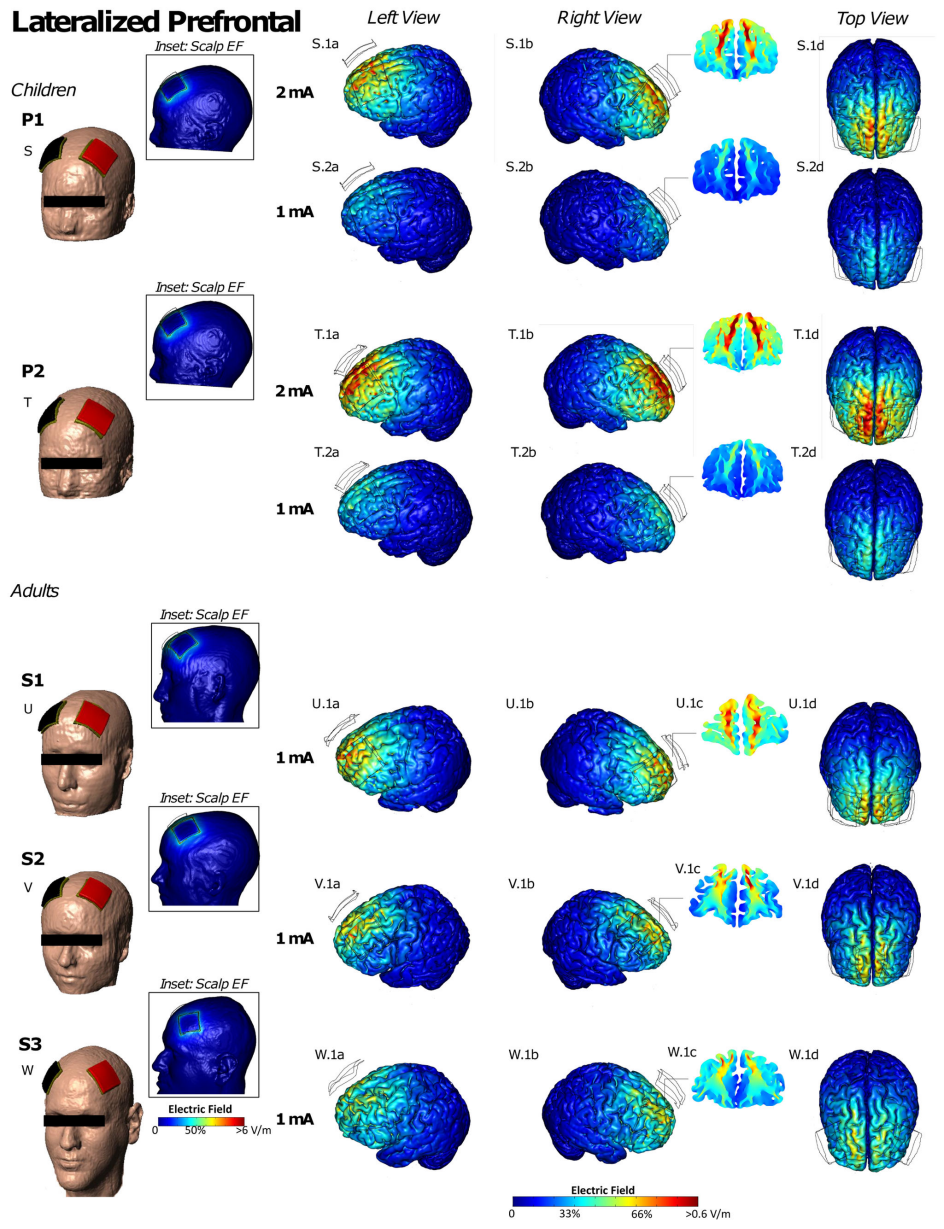
Electric field plots on the cortical surface of the pediatric and adult brains for Lateralized Prefrontal montage. The center of anode (red) was positioned over the left frontal lobe (F3 electrode location in the 10-20 International System) and the cathode (black) was positioned contralateral to the anode (F4). At 2 mA, the peak electric field was 0.29 V/m, 0.34 V/m, 0.29 V/m, 0.27 V/m, 0.25 V/m for P1, P2, S1, S2, and S3 respectively (S.1a,b,d- W.1a,b,d). S.2a-d, T.2a-d - show EF plots at 1 mA, for the pediatric heads. Cross-sectional coronal electric field plots were taken from the center of the anode (S.1-2c, T.1-2c, U.1c-W.1c).

Differences in the magnitude and distribution of electrical fields produced by electrode montages are illustrated in Figures 3-7, and summarized in Table 2. Peak electrical fields for both P1 (8 year old) and P2 (12 year old) were consistently higher (with a magnitude ranging from 1.3 to 4 times), at a given applied current intensity, than for the relatively large adult model (S3) in every montage. However, peak electrical fields in the smaller adults (S1 and S2) were more comparable to the two pediatric models, with electrical field ranges overlapping to a large degree. For example, for both the 4x1 HD-tDCS montage with 5 cm separation between electrodes and the M1-SO conventional montage, the ranking of peak electrical field intensity from highest to lowest was P2, S1, P1, S2, S3. Of note, the rank list by model of peak electrical field intensities does not simply mirror the rank list of any single anatomic measurement presented above (Table 1). As expected, reducing the applied current for the pediatric models (from 2 mA to 1 mA for conventional tDCS; and from 1.5 to 1 and then 0.5 mA for the 4x1 HD-tDCS), reduced the electric field in each brain region (and thus the peak) by the ratio of applied current reduction.

**Table 2:** Electrical field (EF) ranges and peaks, in volts per meter, for each modeled head, by montage.

		**Montage**					
		M1[A]-SO[C]	4x1 HD-tDCS (5 cm separation) [A center]	4x1 HD-tDCS (2.5 cm separation) [A center]	Lateralized Motor C3[A]-C4[C]	Lateralized Temporal TP7[A]-TP8[C]	Lateralized Prefrontal F3[A]-F4[C]
P1	EF Range (C)	0.11-0.27	0.07-0.11	0.04-0.07	0.25-.37	0.13-0.33	0.05-0.29
	EF Range (A)	0.14-0.30	0.14-0.29	0.18-0.21	0.26-0.44	0.19-0.40	0.05-0.26
	EF Peak	0.33	0.29	0.21	0.44	0.50	0.29
P2	EF Range (C)	0.08-0.31	0.07-0.18	0.05-0.13	0.16-0.40	0.22-0.38	0.04-0.30
	EF Range (A)	0.18-0.44	0.23-0.45	0.23-0.33	0.19-0.40	0.21-0.46	0.05-0.34
	EF Peak	0.44	0.45	0.34	0.40	0.46	0.34
S1	EF Range (C)	0.11-0.30	0.13-0.26				0.06-0.23
	EF Range (A)	0.11-0.30	0.13-0.44				0.05-0.22
	EF Peak	0.36	0.44				0.29
S2	EF Range (C)	0.08-0.28	0.11-0.27				0.03-0.20
	EF Range (A)	0.07-0.24	0.14-0.25				0.03-0.20
	EF Peak	0.29	0.24				0.27
S3	EF Range (C)	0.04-0.19	0.03-0.04		0.09-0.18	0.13-0.26	0.06-0.25
	EF Range (A)	0.07-0.20	0.05-0.07		0.05-0.21	0.12-0.33	0.06-0.25
	EF Peak	0.23	0.11		0.21	0.33	0.25

[A] denotes anode and [C] denotes cathode. Detailed descriptions of montages are contained in the text.

Further consideration of electrical field ranges under the anode and cathode regions introduces further complexity in evaluating sensitivity at a given applied intensity across subjects. For conventional (sponge based) tDCS montages, the peak electrical field is often not under an electrode, but in a region between the electrodes. Thus, a subject with a relatively lower peak electrical field may have a relatively higher regional electric field under an electrode of interest than another subject. For example, the sensitivity ranking for the M1-SO conventional tDCS montage for highest to lowest peak electrical field was P2, S1, P1, S2, S3, but the ranking using the highest value under the anode is P2, P1, S1, S2, S3. In contrast, for the HD-tDCS 4x1 montage, the peak electric field was consistently near the anode or in the area defined by the outer cathode ring, with reduced electric fields outside the cathode ring. Thus for HD-tDCS 4x1 the electric field peak or range under the center anode electrode provides a consistent sensitivity ranking across subjects. Reducing the HD-tDCS 4x1 ring circumference in the pediatric heads from 5 cm to 2.5 cm reduced peak electric field values to intensities broadly more comparable to 4x1 HD-tDCS at 5 cm in adults.

### Current flow distribution

**Figure 8:**
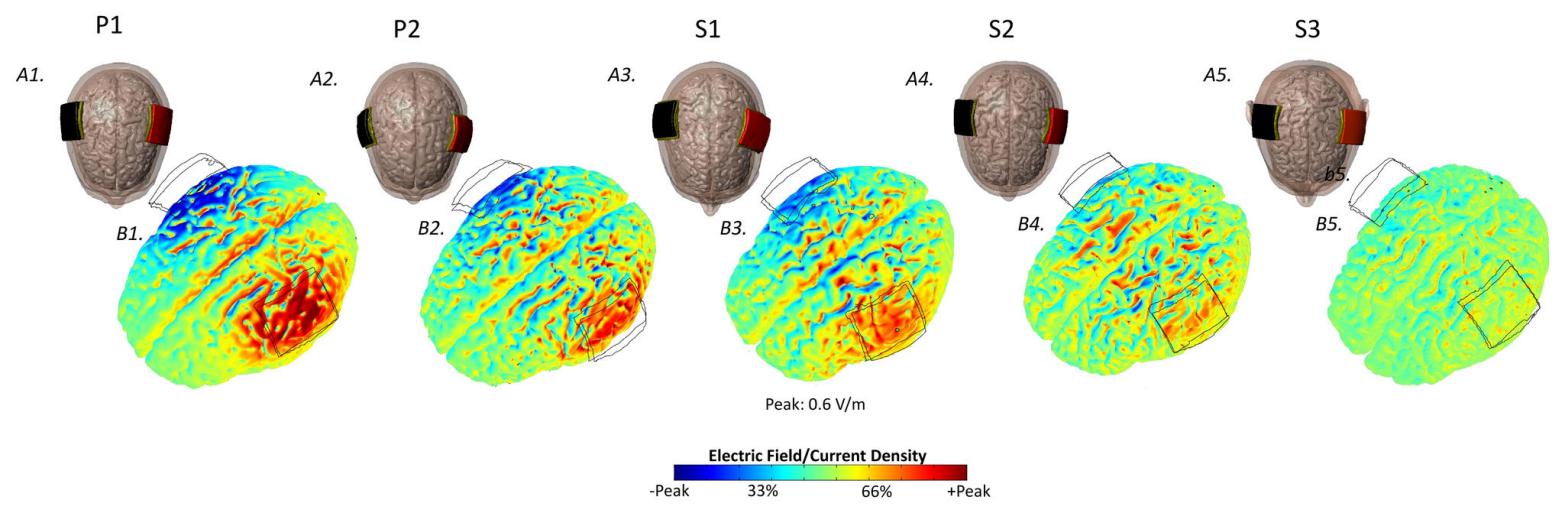
Directionality plots for the lateralized motor montage. The center of anode (red) is positioned on the motor strip and cathode (black) is positioned contralateral to the anode (M-O) (A1-A5). False color map are plotted for 2 mA. The red corresponds to current flowing inwards, the green corresponds to a net flow of zero, and the blue corresponds to current flowing outwards (B1-B5).

As expected, each montage produced a distinct cortical and deep brain current flow pattern. The qualitative patterns of current distribution across the cortex were grossly similar between the pediatric and adult models for specific electrode montages (Figures 3-6). Stimulation with conventional tDCS montages produced current flow under and between the electrodes, and through deep brain structures. Figure 8 illustrates the direction of current flow across the brain, which was comparable between the pediatric and S3 adult head model.

High definition (4x1) montages produce more focal stimulation under the center of the active electrode (in this case the motor cortex unilaterally) with brain current flow largely limited to the cortical region circumscribed by the return electrode ring [8,11–13]. Using a fixed ring diameter of 5 cm, this montage produced a large region of influence in relatively smaller pediatric heads. Reducing the ring diameter to 2.5 cm for the pediatric models restricted the cortical region of influence to an area more comparable to that seen with the larger ring in the adult model.

## Discussion

This is the first study modeling current flow induced by tDCS in pediatric brains. The key prediction of this study is that at a fixed applied current intensity and electrode montage, the range of expected peak electrical fields in a group of children will be higher than in a group of adults. These results have potentially important implications for investigators designing studies utilizing tDCS in pediatric populations.

There is rapidly growing interest in investigating tDCS as a therapeutic modality for brain disorders involving dysfunction of neural networks or impaired cortical excitability [14–18]. During tDCS, weak electrical currents delivered through the scalp modulate ongoing neuronal activity but do not generate action potentials. Low intensity anodal stimulation (current flow inward) is typically expected to increase cortical excitability, while low-intensity cathodal stimulation (current flow outward) is typically expected to decrease excitability [19]. However, the expected direction of change can be reversed by high stimulation intensities, emphasizing the need for vigilance in dosing in experimental design [20].

The safety and tolerability of tDCS, when used within accepted safety guidelines, is well established in adults [21–24]. tDCS has the advantage of being easily portable and inexpensive compared to other noninvasive brain stimulation techniques, such as transcranial magnetic stimulation. Through manipulation of electrode montage, tDCS offers flexibility for targeted delivery of current. The use of tDCS in children has been reported in small case series but the safety of tDCS in children is not yet established, and guidelines on parameters for administering tDCS in pediatric populations have not yet been proposed. Parameters which determine the effects of tDCS not only include current intensity applied at the scalp, electrode locations, and duration of stimulation, but also anatomic considerations, some of which vary with age [16].

### Neuroanatomical and age factors in tDCS

Several neuroanatomic differences between children and adults may contribute to the variance in surface electrical fields observed in this study. The first major anatomic factor to consider is scalp brain distance. Scalp brain distance increases with age, due to increases in skull thickness and extra-axial CSF space [5]. Bone conductivity is low compared to skin or other tissues – therefore, increased skull thickness corresponds with decreased transmission of current from scalp to brain. In contrast, the high conductivity of CSF compared to brain may lead to shunting of current – current that enters into the cranial vault may exit without penetration to the brain surface. Thus, with increased CSF volume (seen with increasing age), greater shunting may result in lowered peak electrical fields in the cortex. The skull thicknesses estimated in our models are consistent with age and sex dependent averages from other anthropologic and imaging studies [25,26]. Our findings are consistent with the prediction that on average, heads with thinner skulls have greater sensitivity to applied current than those with thicker skulls. However, because several anatomic factors, as discussed here, affect dose delivery, peak electrical fields cannot be predicted by skull thickness alone.

Another significant anatomical difference between pediatric and adult brains is the relative proportion of gray and white matter volume. While the greatest increases in total brain volume occur by the age of 5 years, gray matter volume declines by an estimated 1.1% yearly and white matter volume increases by approximately 1.5% yearly from 5 to 18 years of age [4,27]. These differences in gray and white matter content may partially account for the greater depth of current penetration in the pediatric models compared to adult models.

Age-dependent differences in head circumference may also affect current distribution. While the greatest gains in head growth occur in infancy and early childhood, continued growth occurs at a slower pace from middle childhood to adulthood. At age 8 years, the mean head circumference is 52 cm in boys and 51 cm in girls, and at age 18 years, mean head circumferences are 56 and 55 cm in boys and girls, respectively [28]. Thus, conventional tDCS electrodes will cover a larger proportion of the total head area in an average pediatric subject compared to an average adult, leading to a less focal region of stimulation, as illustrated in the lateralized motor montage model (Figure 2). As shown previously in adult models, high definition 4x1 montages produce focused stimulation under the center active electrode (in the case unilateral motor cortex) with current flow largely limited to the cortical region circumscribed under the return electrode ring [11,29]. Compared to conventional electrodes, high definition electrode montages not only have the advantage of increased focality of stimulation, but also the benefit of scalability (Figure 2). By decreasing the distance from the central anode to the return cathode electrodes in the smaller heads, the focality of the electrical fields seen in the adult model using 10-10 system electrode placements is preserved in the pediatric models. These findings are consistent with studies exploring the relationships between electrode area, inter-electrode distance, and the distribution of current density in tDCS [12,30].

Studies of tDCS in adults have generally utilized applied current intensities of 0.5 mA to 2 mA, applied for a period of up to 20 minutes using 5 cm by 5-7 cm sponge wrapped rubber electrodes [31]. Under these parameters, tDCS is safe and tolerable in healthy adult subjects and in those with a variety of neurologic and psychiatric disorders [21–24]. However, the efficacy and risk of tissue damage associated with electrical stimulation is related more closely to current density in the brain (bioavailable dose) than to applied current intensity at the scalp (applied dose). Therefore, extrapolating observations of safety and efficacy in adults may not be appropriate for determining acceptable parameters in children. Our findings suggest that lower applied current intensity (~1 mA) may achieve brain current densities in pediatric subjects on average comparable to densities seen in adults exposed to 2 mA current, but there are significant variations across pediatric and adult heads in sensitivity to a specific applied current, and comparisons are montage specific. Our modeling predictions of current flow do not show that applying 2 mA of current is unsafe in children. However, caution is warranted in applying higher tDCS doses because the average bioavailable dose is likely to be higher in a group of children undergoing tDCS than in a group of adults.

### Empirical experience with tDCS in children

To date there are limited reports in the medical literature of the use of tDCS in children. A small pilot study in children with schizophrenia evaluated the safety and tolerability of tDCS in twelve patients ages 10-17 years [32]. Patients received either bilateral dorsolateral prefrontal cortex anodal stimulation (with an extracephalic cathode), or bilateral superior temporal gyrus cathodal stimulation (with an extracephalic anode) for 10 sessions over 2 weeks. Using 25 cm^2^ sponge electrodes, 2 mA of current was applied for 20 minutes during active sessions and 1 minute for sham sessions. As in most studies in adults, the two most commonly reported side effects of stimulation were tingling (20% of subjects) and itching (40% of subjects). None of the subjects had significant adverse events or side effects, and none withdrew due to effects of tDCS.

A second small open-label pilot study of tDCS for childhood dystonia evaluated a single session of cathodal stimulation over motor cortex, with the anode over the contralateral forehead (M1-SO montage), using 35 cm^2^ sponge electrodes [33]. The ages of study participants ranged from 7 to 18 years (median 13.5 years). One mA of current was applied for 9 minutes, followed by a 20-minute pause and an additional 9 minutes of stimulation. One participant withdrew from the study due to discomfort during stimulation, and a second participant received a reduced dosage (0.65 mA) due to scalp discomfort. Otherwise, there were no other adverse events. Stimulation effectively reduced involuntary overflow of movements in a small subset of participants but not in the overall cohort.

A case was reported of an 11-year-old child with treatment resistant epilepsy who underwent 2 mA cathodal stimulation over the epileptogenic zone using 25 cm^2^ sponge electrodes (cathode over right parietal temporal region and anode over left supraorbital region) [34]. Stimulation duration was 20 minutes daily, five times per week over two weeks. No adverse effects were reported, including no evoked seizures during the stimulation period. A decline in the frequency of seizures was observed in the post treatment period. In a pilot study of tDCS for epilepsy using a sham controlled cross over design, five children with the syndrome of continuous spike wave in sleep received a single 20 minute session of cathodal stimulation at 1 mA using a 25 cm^2^ sponge electrode over the location of peak negativity of epileptiform discharges, and a 100 cm^2^ return electrode over the area of peak positivity [35]. While the outcome of interest (suppression of spike index) was not observed, all subjects, ages 6-11 years, tolerated stimulation without adverse events or apparent side effects. While the stimulation intensity was lower than in two of the other studies discussed here, a large return electrode was used to increase the current density in the target area.

Finally, a randomized controlled trial of 36 children (27 in an active stimulation group and 9 in a sham stimulation group) with treatment resistant epilepsy was recently published [36]. Subjects had a mean age of 11 years in both groups, and ranged from 6 years to 15 years. Epilepsy etiologies were varied, and included focal cortical dysplasia and stroke related injury. Active stimulation consisted of a single session of 1 mA current applied for 20 minutes, with the cathode positioned over the presumed epileptogenic region and the anode over the contralateral shoulder. In the immediate post-stimulation period and at 24 and 48 hours after active stimulation, there was a significant decrease in frequency of epileptiform discharges. Four weeks after treatment, a small reduction in seizure frequency was also detected. Of note, a single adverse event occurred, consisting of transient erythema on the shoulder. Because the study was primarily a safety and preliminary efficacy study, stimulation was limited to one session, with plans to study the effect of multiple sessions in future studies. The studies summarized here suggest that stimulation at intensities between 1 and 2 mA may be tolerated by children, but too little information is available at this point to draw any conclusions about the physiologic effects of stimulation at different intensities in children.

### Toward rational tDCS dose in children

For all investigational therapeutics, dose determination is a key step in treatment development, with early stage trials typically delineating dose-safety and dose-efficacy relationships. Special attention to dose selection is required when therapeutic use or investigation is extended to the pediatric population (a position supported by both the U.S. Food and Drug Administration and European Union regulations) [37]. For ethical and practical reasons, dose selection in children is often based on empirical extrapolation from clinical trials in adults – a practice which has obvious limitations, both for pharmacologic agents and devices delivering electrical or electromagnetic stimulation. Limited empirical studies in children suggest tDCS at stimulation intensities typically used in adults may be well tolerated, but explicit guidance for tDCS dosing in children is lacking. Two key factors influence the need for dose adjustment in children: anatomic differences leading to differences in bioavailable dose, and differences in neurophysiology and disease physiology, leading to differences in dose-efficacy relationships. This study uses computational modeling methods to address the first question – is the bioavailable dose for a specific applied current intensity likely to be different in children than in adults. While the dose-efficacy question remains unclear, the findings presented here can be used as a starting point in developing rational dosing regimens for clinical trials.

**Figure 9:**
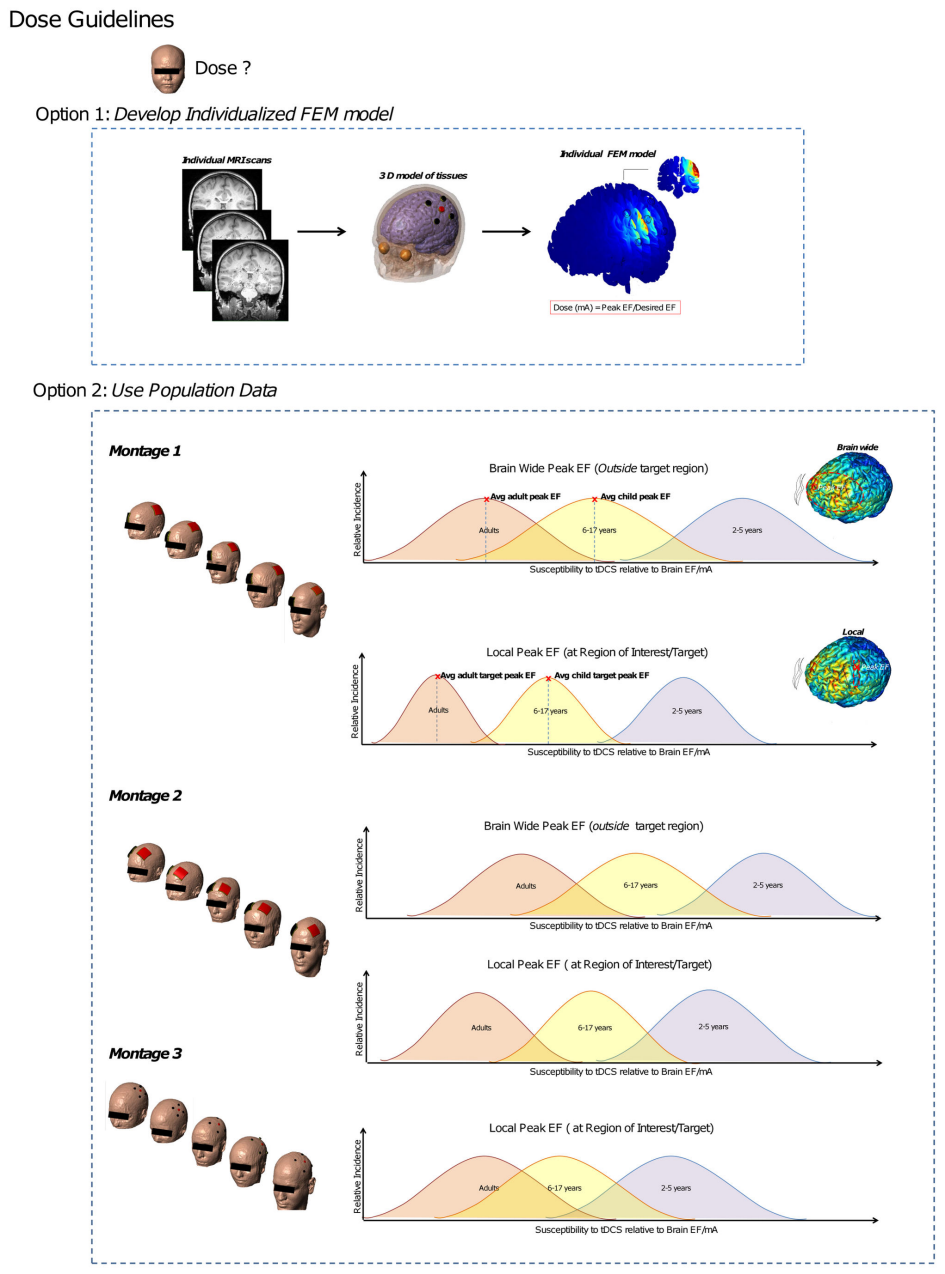
Approaches to normalize dose across populations. Top- Even in cases when individual modeling is practical for every subject in a study, criterion (based on population response) may be rewired to selected desired brain electric field parameters. Bottom: For each given montage and age range, there is a distribution of sensitivity (defines as the electric field in the brain per mA of current applied). In cases where the peak electric field is outside the nominal target (as is typical the case for sponge montages) further consideration should include both brain wide peak electric field and local electrical field maxima inside the nominal target. In the case of 4x1 HD-tDCS, the peak electric field is inside the ring so at the nominal target. When determining a normalized dose for a pediatric population is thus important to recognize scaling will be both montage and age dependent.

The optimal application of computational models requires a clear sense of their constraints [38]. Finite element models are limited by the accuracy of tissue dimensions and conductivity values incorporated. In this study, high-resolution MRI scans were used to incorporate a high degree of anatomical detail into the models to explore differences in normal anatomy between pediatric and adult subjects. A limitation of this study is that only one representative model was used for each of two ages. There may be significant individual anatomic variability affecting the magnitude and distribution of cortical electric fields, and age effects may not be linear through the 8 to 12 year old window we chose to study. Therefore, development of multiple models across a broader range of ages may further inform the choice of optimal parameters for pediatric studies of tDCS (Figure 9). However, this study is the first to address potential differences in tDCS dose requirements in children, and the concerns raised should be considered by any investigators considering studying tDCS in any pediatric population. While models of brains across a broader range of ages will be useful, individualized models for subjects in a therapeutic trial may be possible in the future with further advances in automated methods for segmentation and modeling (Figure 9).

Computational models can provide valuable pre-clinical information to guide decisions about stimulation parameters, but several questions remain about the relationship between regional electrical fields, neuroanatomy, and the physiologic effects of tDCS. As these relationships become clearer, models can be further refined, and the contribution of models to parameter optimization may grow further. For example, while we expect that the greatest effect of tDCS is on brain regions exposed to the highest magnitude of electrical field, factors which determine how neurons within the electrical field are affected are still being elucidated. For example, recent evidence in rat hippocampal slices suggests that the orientation of axons within a constant electrical field influences whether direct current stimulation leads to regional excitation or inhibition [39]. Thus, taking into account the predominant direction of tracts within a stimulated area may improve the usefulness of these models.

Finally, these models do not address the potential for differences in tDCS effects based on variation in cerebral physiology during development compared to a fully matured state. For example, age dependent mechanisms of developmental plasticity may impact the nature and magnitude of the persistent effects of tDCS. For these reasons, modeling studies are not replacements for carefully conducted clinical studies designed to ask specific questions in specific populations. Nevertheless, the information obtained from modeling studies in a target population can be a critically important starting point for designing early phase dose finding and safety studies.

Our models predict that the dose of current seen at the cortical surface after application of tDCS at a specific applied current intensity will be higher on average in children than adults. However, within our limited selection of subjects, the average sensitivity in children (8-12 years) is comparable to more sensitive adults with smaller head sizes. Taken together, our results demonstrate the need for caution in applying 2 mA or greater current intensity in pediatric populations.
